# Proceedings of the Simulation Congress 2021: Virtual. Liège, 22^nd^ October 2021 https://www.simulationcongress.uliege.be

**DOI:** 10.1186/s13690-021-00716-y

**Published:** 2021-12-15

## Evidenced-based Simulation

### A1 Use of Vandenberg and Kuse Mental Rotation Test to Predict Practical Performance of Sinus Endoscopy

#### Florence Rogister^1^, Laurence Pottier^1^, Ilyas El Haddadi^1^, Justine Monseur^2^, Anne-Francoise Donneau^2^, Anh Nguyet Diep^2^, Severine Camby^1^, Valérie Defaweux^3^, Pierre Bonnet^3^, Sophie Tombu^1^, Philippe Lefebvre^1^, and Anne-Lise Poirrier^1^

##### ^1^ Department of Otorhinolaryngology, University Hospital of Liege, Liege, Belgium; ^2^ Biostatistics Unit, Department of Public Health, University of Liege, Liege, Belgium; ^3^ Department of Anatomy, University Hospital of Liege, Liege, Belgium

###### **Correspondence:** Florence Rogister (florence.rogister@chuliege.be)

**Background**

The aim of this study was to assess the predictive value of the Vandenberg and Kuse Mental Rotation Test (MRT) [1] on performance of novice medical students for manipulation of a nasal endoscope on a cadaveric model [2,3]. Regarding spatial abilities, MRT is one of the most widely used [1]. The aim is to identify correct reproductions of a 3-dimensional reference figure.

**Materials and methods**

We randomly selected 39 medical students who had never handled a nasal endoscope and subjected them to the MRT. General information including experience in manual, technical, or surgical activities and testing of anatomical knowledge were collected [4]. They were then asked to perform series of cadaveric model exercises using a nasal endoscope. Their cadaver performance was evaluated by 2 blinded observers. We used an adapted version of the 7-point scale established by Lindquist et al [5,6]. This is a standardized Global Rating Scale using a 5-point Likert-type scale with different dimensions (installation, control of the camera, quality of the exposure, tremors, control of hands movements, quality of the manipulations, and completion of the requested task). The overall score is calculated as the sum of the scores from the 7 subdimensions.

**Results**

We found that medical students with higher MRT score had significantly increased endoscopic sinus performance (P=.0002 using multivariate regression adjusted for specialty choice, previous surgical exposure, and anatomy knowledge). Higher anatomy score was also associated with better endoscopic sinus performance (P=.0141). Other parameters had no impact on endoscopic sinus performance (surgical orientation, previous surgical clerckship, previous sinus surgery experience, surgical assistance experience : P > .05).

**Conclusions**

The score obtained on the MRT was correlated with the practical performance of manipulating the nasal endoscope in cadaver. It could therefore be a useful spatial ability tool for directing targeted training in rhinology.

**Acknowledgements**

The author(s) declared no potential conflicts of interest with respect to the research, authorship, and/or publication of this abstract

**References**

1. Caissie AF, Vigneau F, Bors DA. What does the mental rotation test measure? An analysis of item difficulty and item characteristics. Open Psychol J. 2009, 2, 94-102.

2. Reznick RK. Teaching and testing technical skills. Am J Surg. 1993;165:358-361.

3. Jelovsek JE, Kow N, Diwadkar GB. Tools for the direct observation and assessment of psychomotor skills in medical trainees: a systematic review. Med Educ. 2013;47(7):650-673.

4. Yamauchi Y, Yamashita J, Morikawa O, et al. Surgical skill evaluation by force data for endoscopic sinus surgery training system. MICCAI 2002, LNCS 2488, 44-51;2002.

5. Lindquist NR, Leach M, Simpson MC, Antisdel JL. Evaluating simulator-based teaching methods for endoscopic sinus surgery. Ear Nose Throat J. 2019;98(8):490-495.

6. Laeeq K, Waseem R, Weatherly RA, et al. In-training assessment and predictors of competency in endoscopic sinus surgery. Laryngoscope. 2010;120(12):2540-2545.

### A2 Impact of virtual reality training in disaster medicine

#### Sabrina Chevalier^1,2^, Nadège Dubois^1^, Marie Gontariuk^3^, Alexandre Ghuysen^1,4^

##### ^1^ Department of Public Health, Faculty of Medicine, University of Liege, Liege, Belgium; ^2^ Intensive Care Unit, Clinique Notre-Dame de Grâce de Gosselies, Gosselies, Belgium; ^3^ Faculty of Health, Medicine and Life Sciences (FHML), Care and Public Health Research Institute (CAPHRI), Department of Health, Ethics and Society, Maastricht University, Maastricht, The Netherlands; ^4^ Emergency Department, University Hospital of Liege, Liege, Belgium

###### **Correspondence:** Sabrina Chevalier (Sabrina.Chevalier@uliege.be)

**Background**

Crisis and disastrous events might have various impact in terms of human, material and financial costs [1]. Disaster medicine aims at optimizing resources management which the number of victims is high and resources limited, notably by using triage systems [2]. However, many difficulties persist when it comes to practice triage in real life [3], re-emphasizing the importance to train efficiently the healthcare professionals. In this context, simulation appears to be offer real interests. Within the framework of the INTERREG project "International Knowledge and Information Centre" (IKIC), a virtual reality environment simulating a train accident has been created and funded. We tested the impact of this training on participants’ satisfaction and learning.

**Materials and methods**

This quantitative study included 17 nurses specialised in emergency medical aid. These students participate in our training course which proceeded through several steps: pre-course e-learning, pre-briefing, briefing, virtual reality scenario and debriefing. Questionnaires were distributed before and after the simulation to assess satisfaction, self-efficacy and key learnings. The focus of the training was on the pre-triage of victims.

**Results**

Our training increased participants' sense of self-efficacy (p-value = 0.04). We also noticed high levels of satisfaction and confidence in acquired learning and a high sense of presence. The majority (70.59%) of students felt that the environment had a positive impact on their performance. However, knowledge did not change after the training (p-value = 0.44).

**Conclusions**

In this experience, using a simulation training with a virtual reality environment had a positive impact on participants satisfaction and perceived self-efficacy. The fidelity of the environment was underlined by the participants. In future research, it would be interesting to compare such virtual reality training with conventional training.

**References**

1. Merciefca JM, Ammirati CH, Amsallem C. La formation des infirmiers en médecine de catastrophe. Société française de médecine d’urgence. 2010 ; 1173- 1183.

2. Jouffroy R, Nahon M, Delpech P, Puidupin A, Tourtier J-P, Carli P, Vivien B. Comment organiser les soins pré- et intrahospitaliers en cas d’affluence massive de patients ?. Réanimation. 2015 July; 24: 557-572.

3. Cicero M, Whitfill T, Overly F, Baird J, Walsh B, Yarzebski J, Riera A, Adelgais K, Meckler G, Baum C, Cone D, Auerbach M. Pediatric Disaster Triage: Multiple Simulation Curriculum Improves Prehospital Care Providers' Assessment Skills’. Prehosp Emerg Care. 2017 Mar-Apr;21(2):201-208.

### A3 Effect of skill lab training for intravenous cannulation on performance and satisfaction in undergraduate veterinary students - preliminary results

#### Alexandru Tutunaru^1^, Johann Detilleux^2^, France Beaufays^1^, Stefan Deleuze^1^, Charlotte Sandersen^1^

##### ^1^ Department of Companion Animal Clinics, Faculty of Veterinary Medicine, University of Liège, Liège, Belgium; ^2^ Department of Animal Production, Quantitative Genetics, Faculty of Veterinary Medicine, University of Liège, Liège, Belgium

###### **Correspondence:** Alexandru Tutunaru (actutunaru@uliege.be)

**Background**

The present study aims to demonstrate an improvement of intravenous cannulation skills after skill lab implementation for final year veterinary students.

**Materials and methods**

Previous experience, confidence to place an intravenous (iv) cannula and the success rate of iv cannula placement were evaluated in final-year veterinary students by using a questionnaire. First, the students enrolled in the study filled a questionnaire containing questions to determine previous experience and their personal opinion on the usefulness of mannequin experience, theoretical courses and their confidence to perform the cannulation successfully. Next, all students practiced iv cannulation during clinical rotation and their performance was recorded by auto-evaluation. Results were analysed by Chi-square tests where appropriate.

**Results**

Sixty-one students enrolled in the “non-experienced” group (NonExp), 38 students enrolled in the “clinical experience” (ClinExp) group, 35 students enrolled in the “skill lab group” (SkillLab), and 12 students in the group with experience in both, clinical and skill lab mannequin (ClinSkil). Previous experience was significantly different among groups with 77 % of the NonExp students have placed less than 5 catheters in dogs and 74% of the ClinExp group have placed more than 5 catheters before starting their clinical rotation. Students of all groups felt equally confident to place an iv cannula successfully. In the SkillLab group, 43 % of the students felt that mannequin training helped them placing an iv cannula, while 34% thought that mannequin experience did not help them and 23% had no opinion on this question. Success rate of iv cannula placement during clinical rotation was not significantly different among groups but tended to be higher in students that felt more confident.

**Conclusion**

The preliminary data presented here fails to demonstrate a difference between skill lab and clinical experience in iv cannula placement in final year veterinary students.

### A4 The impact of soft skills on scorpion envenomation management during high-fidelity simulation in nursing education

#### Mohamed Benfatah^1^, Omaima Changuiti^1^, Abdelghafour Marfak^1,2^, Elmadani Saad^1^, Abderraouf Hilali^1^, Ibtissam Youlyouz-Marfak^1^

##### ^1^Hassan First University of Settat, Higher Institute of Health Sciences, Laboratory of Health Sciences and Technologies, Settat, Morocco; ^2^ National School of Public Health, Rabat, Morocco

###### **Correspondence:** Mohamed Benfatah (benfatah.isss@uhp.ac.ma)

**Background**

Technical skills (TS) and non-technical skills (NTS) play a major role in ensuring patient safety [1, 2], this study examines the impact of NTS on the management of the third stage of scorpion envenomation in a high-fidelity health simulation session.

**Materials and methods**

This is a descriptive study that targeted 24 students (30% Male, 70% Female) in a master's degree in advanced health care at the Higher Institute of Health Sciences in Settat (Morocco). The main objective of the simulation session was to evaluate student’s TS and NTS that are crucially important in daily confronted situations by nurses (e.g the scorpion envenomation cases). TS were evaluated based on the national scorpion envenomation management algorithm [3]. The Anaesthetists Non-technical skills scale (ANTS) was used to assess NTS [4].

**Results**

It was found that the high performing groups mastered all modalities of soft skills, TS and NTS were significantly correlated to each other (r²= 0.96, P= 0.01). Also, learners expressed a positive attitude towards simulation-based learning.

**Conclusions**

This study showed that NTS are associated with TS. Mastering NTS is a key component to ensure effective decision-making, and particularly, in the third stage of scorpion envenomation case.

**References**

1. Riem N, Boet S, Bould MD, Tavares W, Naik VN. Do technical skills correlate with non-technical skills in crisis resource management: A simulation study. Br J Anaesth. 2012;109(5):723–8.

2. Peltonen V, Peltonen LM, Salanterä S, Hoppu S, Elomaa J, Pappila T, et al. An observational study of technical and non-technical skills in advanced life support in the clinical setting. Resuscitation [Internet]. 2020;153:162–8. Available from: https://doi.org/10.1016/j.resuscitation.2020.06.010

3. Bencheikh RS, Khattabi A, Faraj Z, Semlali I. Conduite à tenir devant une piqûre de scorpion au Maroc. Ann Fr Anesth Reanim. 2008;27(4):317–22.

4. Fletcher G, Flin R, McGeorge M, Glavin R, Maran N, Patey R. Anaesthetists’ non-technical skills (ANTS): Evaluation of a behavioural marker system. Br J Anaesth. 2003;90(5):580–8.

## Innovations in Simulation

### A5 The complementarity between simulation and OSCE in the management of eclampsia: learning and assessment

#### Omaima Changuiti^1^, Mohamed Benfatah^1^, Abdelghafour Marfak^1,2^, Elmadani Saad^1^, Abderraouf Hilali^1^, Ibtissam Youlyouz-Marfak^1^

##### ^1^Hassan First University of Settat, Higher Institute of Health Sciences, Laboratory of Health Sciences and Technologies, Settat, Morocco; ^2^National School of Public Health, Rabat, Morocco

###### **Correspondence:** Omaima Changuiti (o.changuiti@uhp.ac.ma)

**Background**

Eclampsia is a life-threatening emergency that causes approximately 60,000 maternal deaths per year worldwide [1]. Although global health training is developing with the increasing needs of the population [2]. Simulation has proven its importance as an innovative teaching strategy in the education of health science students [3]. And to asses these students, the Objective Structured Clinical Examination (OSCE) has been known as one of the most credible methods [4].

**Materials and methods**

This study aims to describe the impact of using high-fidelity simulation in the management of eclampsia amongst midwifery students, and to evaluate this impact through OSCE pre and post simulation. To do that, 31 second year midwifery students, were given a diagnostic assessment through the pre-simulation OSCE, followed by a high-fidelity simulation session, and then, the students were benefited from a post-simulation OSCE session for a summative evaluation. The general objective of all OSCE and simulation sessions, was the management of eclampsia crisis. Adapted assessment grids have been developed and validated according to the specific objectives of each OSCE station, and then a well-adapted evaluation grid that includes items describing technical skills, and non-technical skills was mobilized during the simulation session. The total score of all the grids was 20.

**Results**

The average OSCE grid score for all three stations increased from 9.16/20 to 16.2/20 after the high-fidelity simulation session (p<0.001). The results of the simulation session grid show that the mean total score was 14.54±1.12 (Mean technical skills = 6.37/10; Mean non-technical skills = 8.33/10). There was a statistically significant relationship between the technical and non-technical skill scores (P<0.001).

**Conclusion**

The present study joins simulation and OSCE in a common experience presenting the management of eclampsia. The simulation and OSCE must to be strongly integrated in the learning curriculum of midwifery students given their added value in the learning and assessment of midwifery students.

**References**

1. Raney JH, Morgan MC, Christmas A, Sterling M, Spindler H, Ghosh R, et al. Simulation-enhanced nurse mentoring to improve preeclampsia and eclampsia care: an education intervention study in Bihar, India. BMC Pregnancy Childbirth. dec 2019;19(1):41.

2. Kynes JM, Kauffmann R, Walters CB, Sizemore C, Banerjee A. The Preparing Residents for International Medical Experiences (PRIME) Simulation Workshop: Equipping Surgery and Anesthesia Trainees for International Rotations. MedEdPORTAL [Internet]. 2021 [cited June 1, 2021];17. Available from: https://www.ncbi.nlm.nih.gov/pmc/articles/PMC7880254/

3. Akalin A, Sahin S. The impact of high‐fidelity simulation on knowledge, critical thinking, and clinical decision‐making for the management of pre‐eclampsia. Int J Gynecol Obstet. sept 2020;150(3):354‑60.

4. Kolivand M, Esfandyari M, Heydarpour S. Examining validity and reliability of objective structured clinical examination for evaluation of clinical skills of midwifery undergraduate students: a descriptive study. BMC Med Educ. déc 2020;20(1):96.

### A6 Virtual simulation was an alternative device to help nursing students during the first lockdown

#### Guillaume Decormeille^1-3^, Nathalie Huet^1^, Thomas Geeraerts^2-3^

##### ^1^Cognition, Languages, Language, Ergonomics Laboratory 5263 CNRS, University of Toulouse Jean Jaures, Toulouse, Francel; ^2^University Toulouse 3-Paul Sabatier, Toulouse, France; ^3^Institut Toulousain de Simulation en Santé (ITSimS), University Hospital of Toulouse, Toulouse, France

###### **Correspondence:** Guillaume Decormeille (guillaumedecormeille@wanadoo.fr)

**Background**

COVID-19 pandemic has disrupted the real "face to face" clinical simulation which has been exchanged for the virtual "face to face"[1]. Virtual simulation as Screen based simulator (SBS)[2,3] became the new paradigm for nursing students education to ensure pedagogical continuity. What are the benefits of SBS on the motivation, satisfaction, as an additional tool in training, preventing stress or perceived capacity to reinvest theoretical knowledges in clinical practice (CP)?

**Materials and methods**

An online questionnaire was sent to 323 French nursing schools (NS) who had free access to the 9 SBS from Simforhealth society. Students who did not have the benefit of these SBSs were able to view a video presentation so that they could answer the questions based on their perception as compared to students who had a real experience on SBS.

**Results**

Among 323 NS, 220 transferred the survey to their students (68%) and 1600 students participated to the study. Some (n= 237, 14.8%) were excluded for completeness. 870 students (63.8%) did not use SBS: 31(6.6%) for lack of interest, time (n= 92, 22.1%) or information but wanted to use it (n= 368, 42.3%). Both groups using or not SBS were totally agreed to consider SBS as an additional tool (M = 4.71/6, SD= 0.8), no difference was found for motivation and satisfaction to use it F (1.1339) = 0.42, p = 0.52; F (1.1339) = 2.06, p = 0.15 reciprocally. Students who used SBS (n= 471, 95.5%) were estimated their ability to reinvest their knowledges in CP with a high mean score (M= 65/100, SD= 17.1). SBS could reduce stress before CP (n= 1363, χ2 = 18.7, p<0.001).

**Conclusions**

Students who did not have the opportunity to use the SBS during lockdown reported being as motivated and satisfied to use them in initial training as others to reduce their stress prior to CP.

**References**

1. Luctkar-Flude ML-F, Tyerman J. The Rise of Virtual Simulation: Pandemic Response or Enduring Pedagogy? 2021;57:1–2. doi:10.1016/j.ecns.2021.06.008.

2. Foronda CL. What Is Virtual Simulation? Clinical Simulation In Nursing. 2021;52:8. doi:10.1016/j.ecns.2020.12.004.

3. Lioce L, Lopreiato J, Downing D, Chang T, Robertson J, Anderson M, et al. Healthcare simulation dictionary . Agency for Healthcare Research and Quality. 2020. doi:DOI: 10.23970/simulationv2.

### A7 The involvement of patients in simulations: the connection between the educational objective and the application framework

#### Jennifer Foucart^1^, Thierry Pastur^1,2^, Marie Jacquet^2,3^

##### ^1^FSM, ULB, Brussels, Belgium; ^2^ Haute Ecole Libre de Bruxelles Ilya Prigogine, Brussels, Belgium; ^3^ ULB, Brussels, Belgium

###### **Correspondence:** Jennifer Foucart (jennifer.foucart@ulb.be)

**Background**

The patient partnership model of Montreal has revealed new practices towards the acknowledgement of the patient's experiential knowledge. This model can be applied in healthcare, education and research settings [1]. Currently, the term "patient-partner" is not included in the SSH dictionary [2] or among the standards of best practices in simulation [3]. Regarding the simulation, the terms that can be found are the standardized patient, whose behaviour is standardized for summative evaluations, and simulated patient, whose behaviour is more authentic and more flexible to the needs of the learners. These two terms can be used to describe both the patients, but also the actors. For this reason, the naming can therefore lead to confusion when it comes to simulation : should we use patient-partner or actor as a “simulated patient”?

**Case report**

On the basis of the observations of simulation experiments (+/- 50 sessions in 5 years) carried out as part of an initial training programme in medical communication, we have defined the benefits and limitations of using patient-partner or simulated patients in the context of communication simulation. Indeed, in order to use patient-partners in simulation, several criteria should be fulfilled: the training of the patient but also an adequacy between the objective of the simulation and the patient profile. Also, the scenario should not include too many aspects of emotional patient management when involving the patient-partner instead of the simulated patient.

In complex communication situations (bad news announcement), the patient partner can complete the role-play talking about his own experience. His involvement as a simulated patient must be thought through with all partners : teacher, old student, patient partner, simulated patient-actor and simulation trainer.

**Conclusions**

The applications of human simulation are as broad as the objectives they serve, and therefore require better definition of the contours of a partnership with patients.

**References**

1. Pomey MP, Flora L. et al. Le « *Montreal model* » : enjeux du partenariat relationnel entre patients et professionnels de la santé. Santé Publique 2015/HS (S1), pages 41 à 50

2. Lioce L., Lopreiato J. et al. Healthcare Simulation Dictionary. Second Edition 2020. The Society for Simulation in Healthcare: {http://www.ssih.org/Dictionary}

3. INACSL Standards Committee. INACSL Standards of Best Practice: Simulation (SM) - Simulation Glossary. Clinical Simulation Nursing. 2016; 12, S39-S47.

## Teamwork & Simulation

### A8 A first simulation MUST go well : Ice-breaking stress and cross-cutting skills

#### Aurore Strimel, Matthieu Pestiaux

##### Haute Ecole de Louvain en Hainaut, Montignies-sur-Sambre, Belgique

###### **Correspondence:** Aurore Strimel (strimela@helha.be); Matthieu Pestiaux (pestiauxm@helha.be)

**Background**

Health simulation has become essential in many medical and paramedical disciplines. Our experience describes a discovery scenario used to introduce a one-day simulation training which was organized ten times. The goals are to reduce the participants’ stress and to create an atmosphere that encourages discussion and learning during the debriefing [1].

**Materials and methods**

Participants: two actors, the first is playing a physiotherapist supervisor and the second is playing a geriatric patient, they are also the simulation instructors, and four learners taking on their role of interns [2]. Discovery scenario: This scenario takes place in a private hospital room, in a geriatrics or pneumology department. The physiotherapist supervisor takes his trainees to put a patient back to bed and to administer an aerosol in a context of swallowing disorders and dementia. The supervisor is not focused on the situation; the patient is disoriented and uncompliant with the instructions. The supervisor lets the learners deal with the patient and doesn’t give them any support [3]. During the debriefing, the following topics are discussed: hierarchical relationship and its impact on stress and reflexivity. Finally, the impact on the stress level is assessed by the feeling expressed by the participants before and after the simulation.

**Results**

The adjectives named by participants show that the stress has been reduced (before vs after): stress vs reassured, nervous vs comprehension of what is expected, worry vs impatient to discover the next simulation.

**Conclusions**

We observed that our discovery scenario has allowed the students to reduce their stress, to become involved in the simulation, and it has promoted communication and group cohesion. In order to further assess the stress level of this scenario on the learners, it would be interesting to conduct a study on this subject.

**References**

1. Gonzalez-Caminal, G., Camps, A., Lorza, G., & Jubany, J. (2018). Simulation as an experience to prepare physiotherapy students for clinical practice - Preliminar perspectives at faculty of Health Sciences at Manresa. 12th Internaional Technology, Education and development Conference. Valencia. doi:10.21125/inted.2018.1614

2. Kameg, K. M., Szpak, J. L., Cline, T. W., & Mcdermott, D. S. (2014, November). Utilization of Standardized Patients to Decrease Nursing Student Anxiety. Clinical Simulation in Nursing, 10(11), pp. 567 - 573. doi:http://dx.doi.org/10.1016/j.ecns.2014.09.006.

3. Nielsen, B., & Harder, N. (2013, November). Causes of Student Anxiety during Simulation: What the Literature Says. Clinical Simulation in Learning, 9(11), pp. e507 - e512. doi:http://dx.doi.org/10.1016/j.ecns.2013.03.003

## Quality & Security of care

### A9 Covid19 crisis as a trigger to rethink hospital management and organization context: a mixed methods study

#### Méryl Paquay^1,2^, Zoé Kabanda^1^, Anh Nguyet Diep^3^, Alexandre Ghuysen^1,2^

##### ^1^Emergency Department, University Hospital of Liège, Liège, Belgium; ^2^Public Health Department, Faculty of Medicine, University of Liège, Liège, Belgium; ^3^Public Health Sciences Department, University of Liege, Liege, Belgium

###### **Correspondence:** Méryl Paquay (meryl.paquay@chuliege.be)

**Background**

Many studies have addressed the consequences of public health crises [1,2]. However, very few authors have explored the impact of these crises on hospitals organization. The aim of this study was to identify changes in the organizational and managerial context in the hospital environment following the Covid crisis.

**Materials and methods**

A mixed methods approach with a convergent design was adopted. For the quantitative phase (n=98), the validated COMEt questionnaire was administered to a convenience sample of physicians, nurses and caregivers to measure the managerial and organizational context before and after the crisis. For the qualitative phase (n=102), a convenience sample of the same healthcare workers and managers were recruited to attend interviews. Questions were asked about the hospital crisis management using the plus/delta method. Data were then integrated through a data transformation model.

**Results**

Results showed multiple tendencies following the first wave of the Covid crisis. These changes may have impacted Covid and Non-Covid units differently. Interprofessional relationships tended to improve (β = 0.062, p = 0.042, η2p = 0.025), as well as relationships with managers (β = 0.091, p = 0.010, η2p = 0.035). Interviewees stated a fracture between lower and top-level management. Staff satisfaction (β = -0.270, p < 0.001, η2p = 0.092) and perceived performance of the unit (β = -0.273, p < 0.001, η2p = 0.175) also seemed to decrease while burnout was felt higher after the crisis (β = -0.361, p = 0.001, η2p = 0.056). Although there is no consensus, the results highlighted a better crisis management and resilience among Covid units compared to Non-Covid (β= -0.323, p = 0.015, η2p = 0.030).

**Conclusions**

The Covid crisis seemed to have changed the hospital organizational and managerial context. This experience should encourage improved communication within hospitals management levels and better team spirit. The multidisciplinary approach should also be given more consideration to improve work environment to speak as teams rather than functions.

**References**

1. Tisdell CA. Economic, social and political issues raised by the COVID-19 pandemic. Econ Anal Policy 2020;68:17–28. doi:10.1016/j.eap.2020.08.002.

2. Pak A, Adegboye OA, Adekunle AI, Rahman KM, McBryde ES, Eisen DP. Economic Consequences of the COVID-19 Outbreak: the Need for Epidemic Preparedness. Front Public Heal 2020;8:19. doi:10.3389/fpubh.2020.00241.

